# Cardiomyocyte Aldose Reductase Causes Heart Failure and Impairs Recovery from Ischemia

**DOI:** 10.1371/journal.pone.0046549

**Published:** 2012-09-27

**Authors:** Ni-Huiping Son, Radha Ananthakrishnan, Shuiqing Yu, Raffay S. Khan, Hongfeng Jiang, Ruiping Ji, Hirokazu Akashi, Qing Li, Karen O'Shea, Shunichi Homma, Ira J. Goldberg, Ravichandran Ramasamy

**Affiliations:** 1 Department of Medicine, Columbia University Medical Center, New York, New York, United States of America; 2 Department of Medicine, New York University School of Medicine, New York, New York, United States of America; Bristol Heart Institute, University of Bristol, United Kingdom

## Abstract

Aldose reductase (AR), an enzyme mediating the first step in the polyol pathway of glucose metabolism, is associated with complications of diabetes mellitus and increased cardiac ischemic injury. We investigated whether deleterious effects of AR are due to its actions specifically in cardiomyocytes. We created mice with cardiac specific expression of human AR (hAR) using the α–myosin heavy chain (MHC) promoter and studied these animals during aging and with reduced fatty acid (FA) oxidation. hAR transgenic expression did not alter cardiac function or glucose and FA oxidation gene expression in young mice. However, cardiac overexpression of hAR caused cardiac dysfunction in older mice. We then assessed whether hAR altered heart function during ischemia reperfusion. hAR transgenic mice had greater infarct area and reduced functional recovery than non-transgenic littermates. When the hAR transgene was crossed onto the PPAR alpha knockout background, another example of greater heart glucose oxidation, hAR expressing mice had increased heart fructose content, cardiac fibrosis, ROS, and apoptosis. In conclusion, overexpression of hAR in cardiomyocytes leads to cardiac dysfunction with aging and in the setting of reduced FA and increased glucose metabolism. These results suggest that pharmacological inhibition of AR will be beneficial during ischemia and in some forms of heart failure.

## Introduction

Both non-ischemic and ischemic heart failure are associated with a dramatic reduction in fatty acid (FA) usage by the heart. However, despite the need for less oxygen to generate ATP, reducing FA oxidation in non-ischemic human heart failure may not be beneficial. [1] In some animal models of afterload-induced heart failure greater dietary fat intake improves function. [2] Even in acute ischemia, shifting metabolism towards greater glucose has not uniformly improved outcome. [3] It is possible that excess glucose might be directed towards aberrant metabolic pathways. One such pathway is that mediated by aldose reductase (AR). In this context we and others have demonstrated that glucose flux via AR is enhanced under conditions of ischemia-reperfusion [4], [5], [6] and diabetes. [7]

AR is a widely expressed aldehyde-metabolizing enzyme that catalyzes the NADPH-dependent conversion of glucose to sorbitol, the first step in the polyol pathway of glucose metabolism. [8] In this pathway both NADPH and NAD^+^ are consumed as cofactors for the enzymes AR and sorbitol dehydrogenase (SDH). AR flux-driven osmotic stress due to accumulation of sorbitol and oxidative stress due to changes in the ratio of NADPH/NADP^+^ and NAD^+^/NADH may mediate several diabetes complications. [9], [10], [11], [12], [13] Several studies show that AR inhibition reduces complications of diabetes in the lens, kidney, and peripheral nerves. [9], [10], [11], [12], [13]

Increased glucose flux via AR may also cause or accelerate various cardiac diseases. In the setting of myocardial ischemia-reperfusion, human AR (hAR) expressing mice had greater injury, which was prevented by AR inhibition. [4] hAR expression increased atherosclerosis in diabetic mice [14], [15] and its inhibition was also protective. [15] Mice normally have much lower levels of AR than humans. [4], [16] Transgenic AR expression has been proposed to “humanize” the mouse hear t [4], [16] and reproduce actions of AR found in man. In contrast inhibition of the low levels and activity of AR expressed in non-transgenic mice may be toxic to blood vessels. [17]

Two issues pertinent to AR actions and heart function are unresolved. 1) Are the cardiomyocytes within the heart involved in its deleterious actions during ischemia-reperfusion? 2) Are AR's actions most evident in the presence of greater cardiac glucose flux? The heart is the most energy-demanding tissue of the body and under normal physiologic conditions the heart utilizes FA as its chief energy substrate for ATP generation. Whether greater flux of glucose, an AR substrate, will increase pathological effects of AR had not been tested. For that reason, we created mice with cardiac specific expression of hAR using the α–myosin heavy chain (MHC) promoter and studied these animals during aging. In addition, we used two conditions, ischemia reperfusion and genetic reduction of FA oxidation, to test whether hAR effects are most pronounced during greater glucose use.

## Methods

### Animal studies and generation of MHC-hAR/Ppara^−/−^ mice

Creation of the mice and all metabolic and genetic studies were reviewed and approved by the Institutional Animal Care and Use Committees of Columbia University and New York University. A transgenic construct containing a 1.3-kb hAR cDNA was cloned downstream of the α-myosin heavy chain (MHC) promoter (5.4 kb). Nae1 and Pme1 digestion of MHC-hAR produced a linear 7.3-kb fragment that was used for microinjection. Transgenic mice were produced by microinjection of the MHC-hAR construct into fertilized 1-cell C57BL/6×CBA F1 eggs. Transgenic mice expressing hAR were identified by analysis of genomic DNA with primer A (5′ CTGTGTTTCTTGCCTCAT-3′); a sense primer specific to MHC promoter exon 2 and antisense primer (5′- CCGTTAGTGGCACTATTT-3′) specific to hAR cDNA nucleotides. MHC-hAR mice were then crossed twice into the peroxisomal proliferator- activated receptor knockout (*Ppara^−/−^*, Jackson Laboratory) background, resulting in MHC-hAR/*Ppara^−/−^* offspring (Supplementary Figure S2).

### Western blot analysis

Fresh heart tissues from 6- and 12-month-old MHC-hAR mice and their littermate controls were obtained. Total proteins were isolated using RIPA buffer according to the manufacturer's instructions (sc-24948; Santa Cruz Biotechnology Inc, CA). Thirty micrograms of total proteins were subjected to Western blot analysis with following antibodies: hAR and PPARα (Santa Cruz Biotechnology Inc, CA), pyruvate dehydrogenase kinase 4 (PDK4) and BAX (Abcam Inc, MA). For control of protein loading, the blots were stripped and reprobed with mouse tubulin antibody (Abcam Inc; MA). Bands were quantified by densitometry using Molecular Analysis Software (Bio-Rad, CA).

### Heart and plasma lipids

Blood from 6-hour fasted mice was collected for the measurement of plasma total cholesterol (TC), triglyceride (TG), free FAs (FFA), and glucose. TG and TC were measured enzymatically using an Infinity kit (ThermoFisher Scientific Inc. VA) and FFA were measured by a NEFA kit (Wako Pure Chemical Industries, VA). Plasma glucose was measured by Autokit Glucose (Wako Chemicals, VA).

### Quantitative real-time (RT)-PCR analysis

Total RNA was prepared using a Pure Link Micro-to-Midi Total Purification System kit (Invitrogen, CA). One microgram of RNA was initially treated with DNase I for 15 minutes. The RNA samples were then reverse transcribed using the ThermoScript RT-PCR Kit (Invitrogen, CA). Quantitative RT-PCR (qRT-PCR) was performed using an ABI 7700 (Applied Biosystems, CA). Amplification was performed using SYBR Green PCR Master Mix (Applied Biosystems, CA). Primers used for PCR amplification are listed in Table S1. Analysis was performed using Sequence Detection Software (Applied Biosciences, USA). Standard curves were generated using undiluted and diluted (1∶10, 1∶100, and 1∶1,000) cDNA samples from heart tissue. Correlation coefficients were 0.98 or greater. Data were normalized with 18S rRNA.

### Histological analysis

Neutral lipids were assessed in hearts taken from 18-hour-fasted male mice perfused with PBS. The hearts were embedded in Tissue-Tek Optimal Cutting Temperature compound (Sakura Finetek U.S.A Inc. CA). Midventricular sections of myocardium (6 µM in thickness) were stained with oil red O and counterstained with hematoxylin. We used dihydroethidium (D23107; Invitrogen) to examine the superoxide in the frozen heart tissue section. To assess interstitial fibrosis, mouse hearts were perfused with 10% buffered formalin solution. Hearts were subsequently immersed in 10% buffered formalin for 24 h, embedded in paraffin, and 5-µm sections of the ventricles were cut. Sections were stained with Masson's Trichrome.

### TUNEL staining

Cardiac ventricular tissues from 12-month-old MHC-hAR, MHC-hAR/*Ppara^−/−^* and their littermate controls were fixed in formalin, embedded in paraffin, and sectioned. Tissues were stained for DNA fragmentation by a TUNEL protocol according to the manufacturer's specifications (R&D Systems, MN).

### Echocardiographic analysis

Mice were anesthetized with isoflurane and 2-dimensional echocardiography was performed using techniques described previously (Sonos 5500 system; Philips Medical Systems, MA). [18] Echocardiographic images were obtained and recorded in a digital format. Images were than analyzed off-line by a researcher blinded to the murine genotype. Left ventricular end-diastolic dimension (LVDd) and left ventricular end-systolic dimension (LVDs) were measured. Percent fractional shortening was calculated as: %FS = ([LVDd−LVDs]/LVDd)×100.

### Glucose uptake

Sixteen-hour-fasted MHC-hAR mice and littermates were injected intravenously with 1×10^6^ decays per minute (DPM) of 2-deoxy-d-[^3^H] glucose (PerkinElmer). Blood was collected at 0.5, 5, and 20 min after injection. At 60 min, mice were perfused with 10 ml of cold PBS, hearts were harvested, flash frozen in liquid nitrogen, and stored at −80°C. Radioactivity was determined in 10 µl of plasma and 100 µl of heart homogenate on a LS 6500 multipurpose scintillation counter (Beckman Coulter, CA) for the ^3^H counts. Amounts of glucose injected were adjusted by plasma radioactivity counts at 30 seconds after each injection and were compared with plasma counts at the end of the experiments. Tissue uptake was normalized to the respective 30-s plasma counts (injected dose) and compared with control mice.

### Heart tissue fructose content

Cardiac fructose content was measured using the Fructose Assay kit according to the manufacturer's instructions (BioVision, CA). Heart tissues were homogenized in the assay buffer and centrifuge to remove insoluble material. 50 µl of the reaction mix was added to each well containing the fructose standard and test samples, and all wells were incubated for 1 hour at 37°C. The glucose background was subtracted by doing a control without Fructose Converting Enzyme in the reaction. O.D. 570 nm was measured in a micro plate reader.

### Ischemia-reperfusion studies

Surgical procedures relating to coronary artery ligation were carried out as previously described. [19] Left anterior descending (LAD) artery was ligated and after 30 minutes of ischemia the LAD blood flow was restored. Heart samples from the affected ischemic area were further evaluated at 48 hours of reperfusion. Sham treated animals subjected to anesthesia and surgical procedure without occluding LAD were included. TTC staining and Evan's blue were used to determine area at risk/infarct area. [20] Infarction area and the total area were evaluated using Axiovision 4.0 software. Cardiac function was evaluated by echocardiography. Both Pre and post echo measurements were done on isoflurane anesthetized mice as described above. Caspase 3 activity measurements in cardiac extracts and hydrogen peroxide generation in the isolated mitochondria were performed as published earlier [21]

### Statistics

We analyzed data using the Prism software package (GraphPad Software, CA). Comparisons between two groups were performed using unpaired 2-tailed Student's t tests. All values are presented as mean ± SD. Differences between groups were considered statistically significant at P<0.05.

## Results

### Creation of MHC-hAR transgenic mice

**Figure 1:**
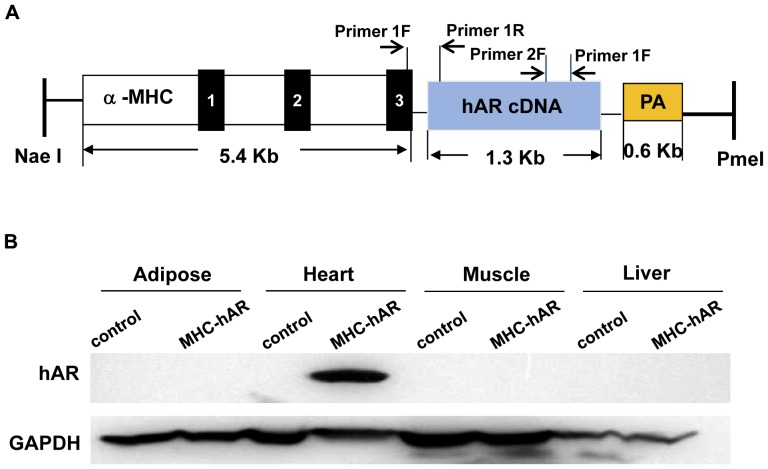
Construct and hAR protein expression in MHC-hAR mice. (**A**) hAR construct design employed in this study is presented. The α-MHC promoter was used to drive hAR cDNA expression. The construct scheme indicate exons that are numbered (black boxes) along with poly(A) site (PA). (**B**) Cardiac-specific hAR expression are presented for. Western blotting was performed in 3-month-old MHC-hAR male mice using polyclonal human AR antibody. Total protein (30 µg) from adipose, heart, muscle and liver tissues of MHC-hAR and their littermate controls was analyzed. GAPDH antibody is shown as a control.

To test whether hAR expression in cardiomyocytes would affect heart function, we created MHC-hAR transgenic mice using the construct shown in Figure 1A. This construct led to high level expression of hAR protein (Figure 1B). Cardiac overexpression of hAR did not alter circulating lipids and glucose levels in 3- and 12-month old mice (Table S1). In 3-month old MHC-hAR mice, heart glucose uptake and mRNA levels of glucose and FA metabolism related genes, such as Glut1, Glut4, CPT1 and AOX, were similar to those of controls. Sorbitol dehydrogenase (SDH) mRNA, which converts sorbitol to fructose, was increased (Figure S1A). Heart/Body weight ratio and cardiac function, as assessed by echo cardiography, was unaffected in the 3 month MHC-hAR mice (Supplementary Figure S1B–D).

### MHC-hAR influences outcome after ischemia reperfusion

**Figure 2:**
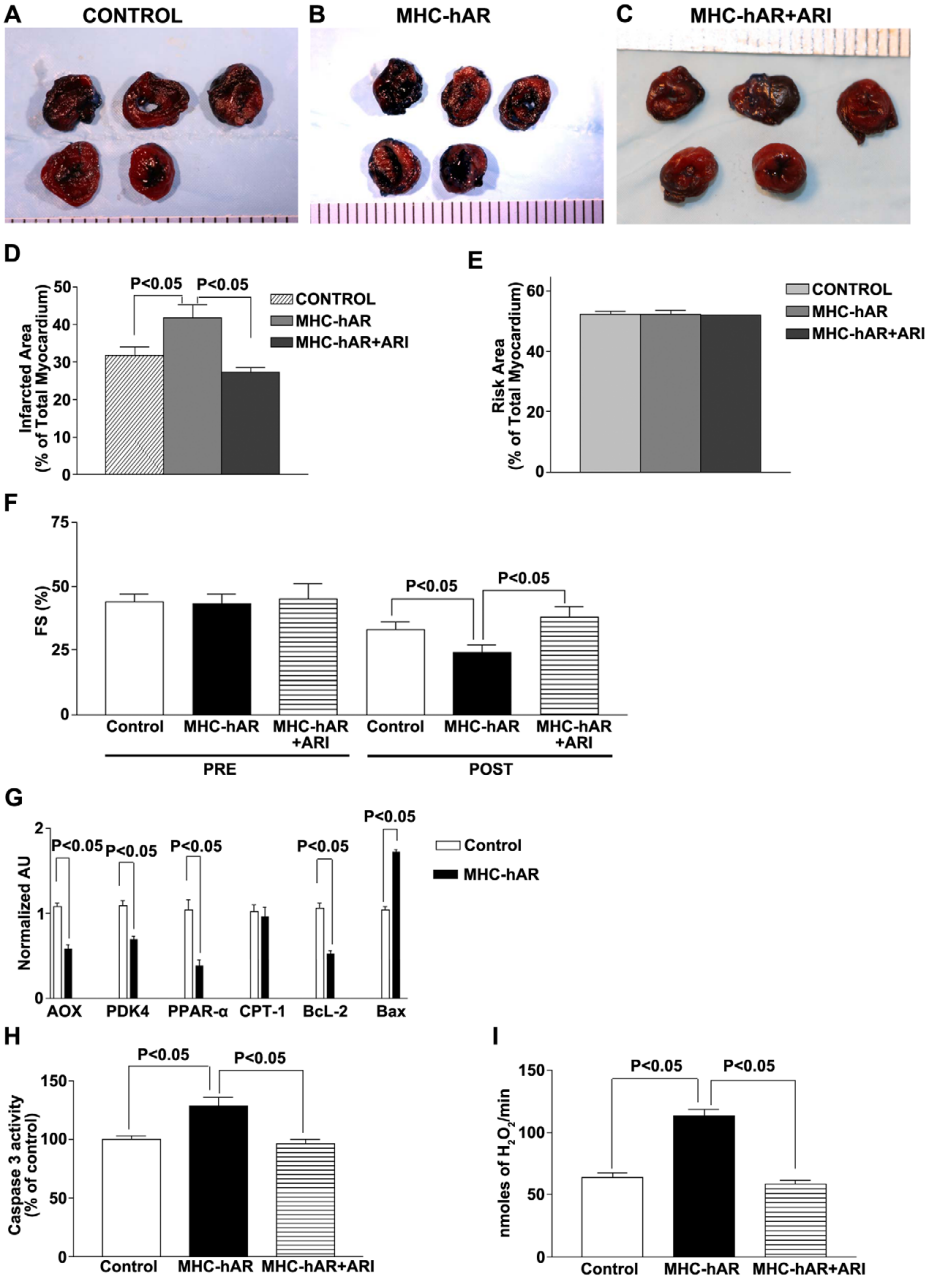
Impact of myocardial infarction in MHC-hAR mice. Hearts were retrieved after LAD ligation followed by 48 hrs of reperfusion and subjected to TTC staining (A–C) for the analysis of infarcted area (**D**) and total risk area in the myocardium (**E**) were determined in the following groups of 3 months old mice: Control Wild type littermates, MHC-hAR, MHC-hAR treated with ARI. (**F**) Measurements of cardiac function using echocardiography were performed prior (Pre) and post LAD ligation. Changes in %fractional shortening (FS) are reported for each group of mice studied. N = 6–8 mice per group. Changes in cardiac FA gene expression in MHC-hAR and littermate controls are presented in **G.** Caspase 3 activities, presented as % of control, are shown for hearts at the end of reperfusion period (**H**). Measurements of hydrogen peroxide released from the isolated mitochondria obtained from MHC-hAR and littermate controls are shown in **I**.

We had previously shown that generalized hAR-expressing mice had worse cardiac function and injury after ischemia due to increased ROS generation and increased apoptosis. [4], [21] To determine if cardiomyocyte hAR mediates ischemia-reperfusion injury, we subjected 3 month-old WT, hAR, and hAR mice-treated with an AR inhibitor (ARI) zopolrestat to LAD occlusion followed by reperfusion. Histologic assessment of heart injury evaluated by TTC staining (Figure 2A–C) revealed that the infarcted area was greater in hAR hearts compared to WT (Figure 2C). Risk area (% of total area) remained equivalent in all groups at about ∼50% (Figure 2D). Infarct area was significantly greater in hAR mice hearts compared to those in WT mice (43.2±3.6% in hAR vs. 31.4±2.7% in WT, p<0.05). Treatment of hAR mice with zopolrestat reduced infarct area compared to those observed in hAR mice (26.7±3.2% in hAR+ARI vs. 43.2±3.6% in hAR, p<0.05). Though the ARI treated hAR mice had less infarct area than untreated WT mice, the data did not reach statistical significance. Heart rates were not altered in different groups of mice studied. FS% was worse in hAR vs. WT mice (Figure 2E) and again ARI treatment of hAR transgenic mice improved functional recovery. LAD ligation/reperfusion in hAR mice was associated with decreased mRNA levels of AOX (p<0.05), PDK4 (, p<0.05) and PPARα (, p<0.05), but not CPT1 (Figure 2F). These data are indicative of hAR linked downregulation of metabolic genes as a key contributor of increased ischemia-reperfusion injury.

Anti-apoptotic gene Bcl-2 was significantly decreased (p<0.05), whereas proaopototic Bax was significantly increased (p<0.05) in hAR mice in comparison with WT mice (Figure 2G). Furthermore, proapoptotic caspase 3 activity was also significantly increased by LAD ligation/reperfusion in hAR mice (Figure 2 H). Mitochondria isolated from hAR mice hearts subjected to LAD ligation/reperfusion exhibited significantly greater generation of H2O2 (figure 2 I) than the mitochondria from littermate hearts. Inhibition with an ARI reduced mitochondrial H2O2 generation in hAR mice. These data indicate that increases in injury due to cardiomyocyte hAR are linked to changes in apoptotis and reactive oxygen species generation.

### MHC-hAR mice develop heart dysfunction with aging

**Figure 3:**
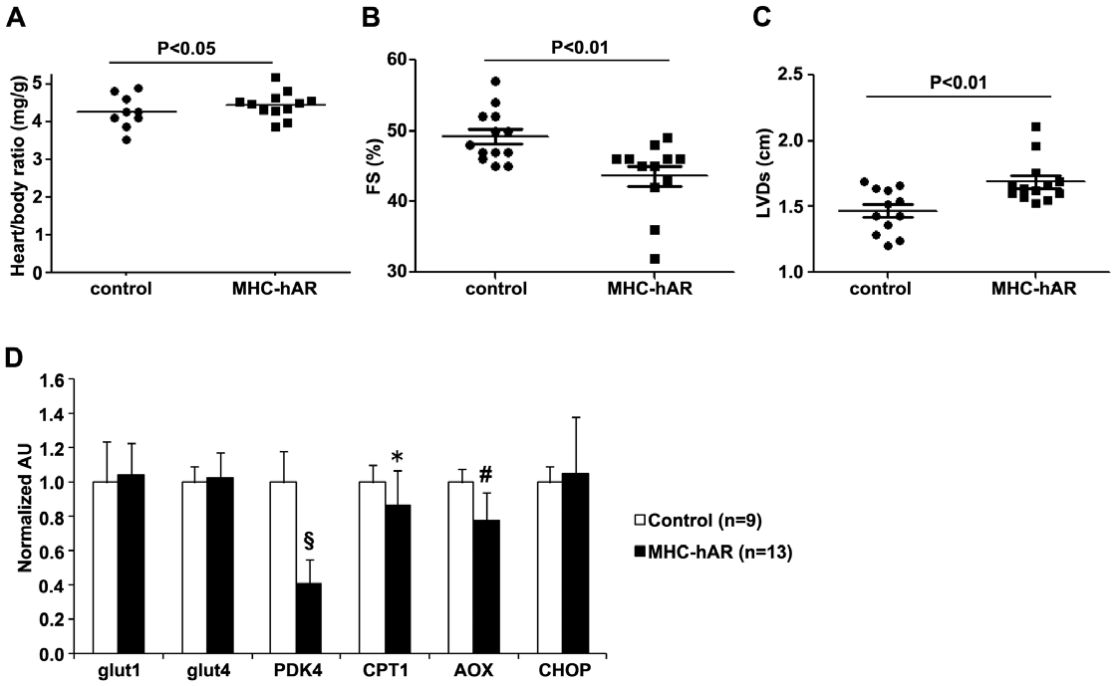
Cardiac dysfunction and reduced FAO related mRNA expression with aging in MHC-hAR mice. 13 month old mice were used in these studies. (**A**) The heart to body weight ratio was increased in MHC-hAR mice (n = 9–12). (**B and C**) Echocardiographic measurements showed increased left ventricular systolic dimension and reduced fractional shortening with age in MHC-hAR mice. (**D**) MHC-hAR transgene altered cardiac FA and glucose oxidation gene expression in mice. FS, fractional shortening; LVDs, left ventricular end-systolic dimension. Data are shown as mean ± SD. *P<0.05, ^#^P<0.01, and ^§^P<0.001 versus littermate controls.

With age, hearts shift to greater glucose and reduced FA oxidation, [22], [23] a change similar to that which occurs during ischemia. To determine whether this change, which results in greater substrate for AR, leads to heart dysfunction echocardiography was performed every three months. Heart function was relatively normal in 3 month-old MHC-hAR mice (Figure S4). However, at 12-months of age MHC-hAR transgenic mice had slightly, but significantly, increased heart/body ratio, reduced %FS and increased LVDs (Figure 3 A–C). These changes in heart function were associated with a decrease in expression of AOX and CPT1 (Figure 3D). Thus, it is likely that, as had been reported in other models of heart failure, the MHC-hAR hearts reduced their use of FA.

### MHC-hAR expression leads to more severe heart dysfunction on the Ppara^−/−^ background

**Figure 4:**
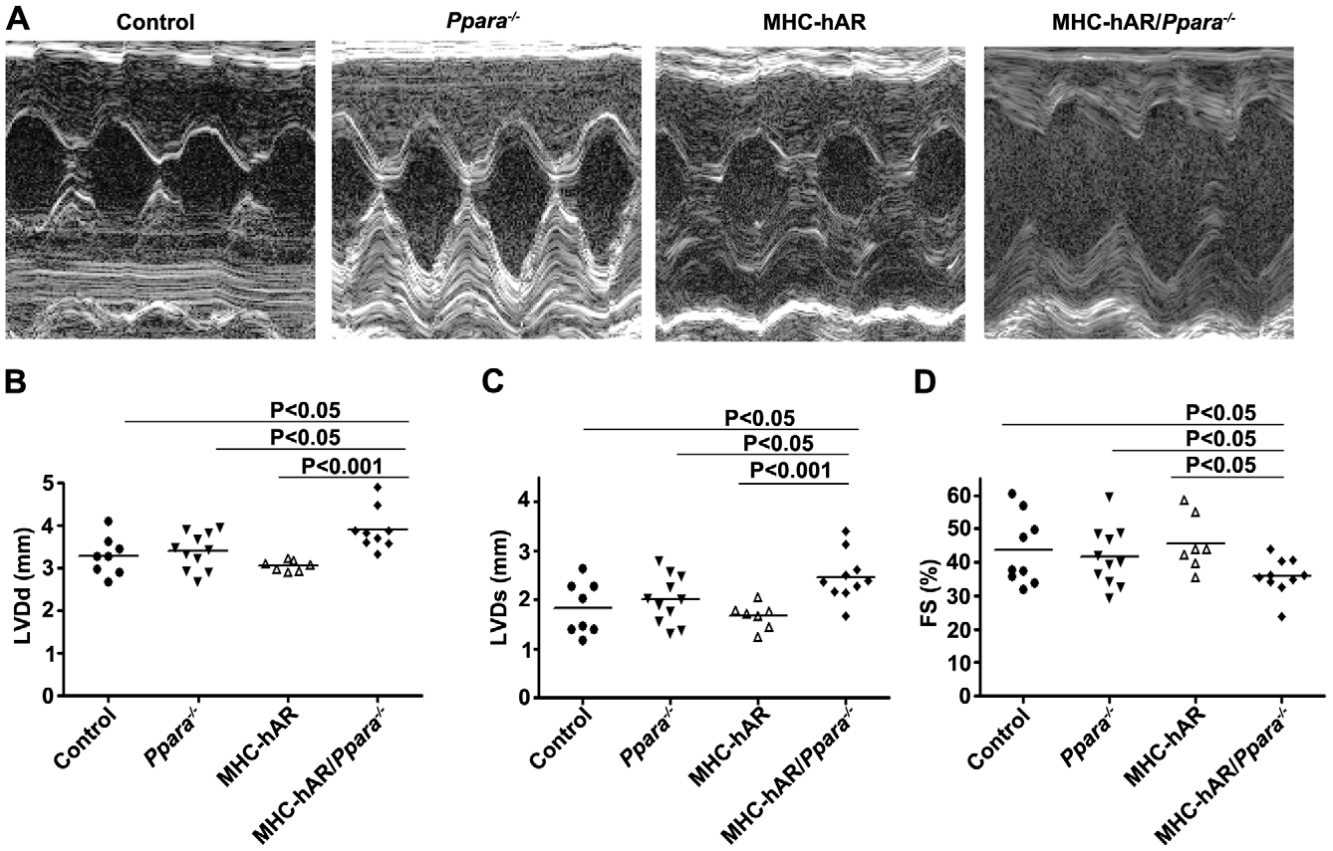
Cardiac dysfunction is observed at an earlier age in MHC-hAR/*Ppara^−/−^* mice. (**A**) Representative echocardiographic images of LVD in the mice (age = 7 months). (**B–D**) Echocardiography showed increased LVDs, LVDd and FS in MHC-hAR/*Ppara^−/−^* mice. FS, fractional shortening; LVDs, left ventricular end-systolic dimension. Data are shown as mean ± SD. *P<0.05 and ^§^P<0.001 versus littermate controls.

We postulated that non-ischemic hearts with a greater requirement for glucose oxidation would also have more dysfunction with hAR expression. To test this, we crossed the MHC-hAR transgene onto the *Ppara^−/−^* background (Figure 4A, Figure S2); these mice have reduced FA oxidation and increased glucose utilization. [24], [25] Plasma FA levels in *Ppara^−/−^* mice were increased, but plasma TG, TC and glucose from each line were similar to those of WT mice (Table S2). To determine if cardiac glucose uptake was increased in MHC-hAR/*Ppara^−/−^* mice, we used 2-deoxy-d-[^3^H]-glucose to assess myocardial glucose import *in vivo*. Plasma clearance of the tracer did not differ between the genotypes. Cardiac glucose uptake was increased 2.1-fold in the MHC-hAR/*Ppara* ^−/−^ mouse hearts compared with the controls (P<0.05; Figure S3A, B). MHC-hAR/*Ppara^−/−^* mice exhibited reduced %FS and increased LVDd and LVDs compared with WT controls, MHC-hAR and *Ppara^−/−^* mice (Figure 4A–D).

### MHC-hAR expression alters metabolic gene expression on the Ppara^−/−^ background

**Figure 5:**
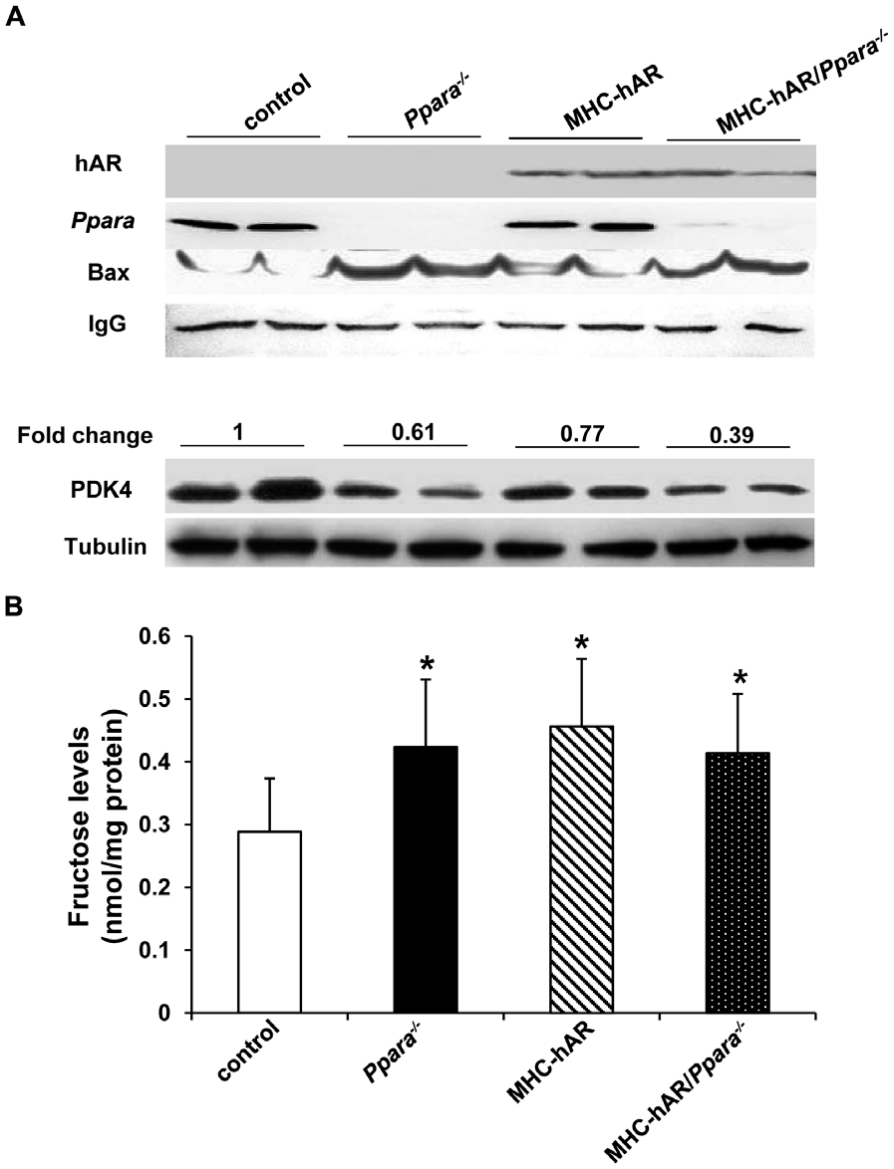
Cardiac fructose content and proteins expression in MHC-hAR*/Ppara^−/−^* mice. (**A**) Representative Western blot images of hAR, PPAR α, PDK4 and BAX proteins in the heart from all the groups of mice studied. IgG bands are shown as control. (**B**) Cardiac fructose content (nmol/mg protein) in MHC-hAR/*Ppara* ^−/−^ mice are compared to those in MHC-hAR mice. Data are shown as mean ± SD (n = 5–7). *P<0.05 versus littermate controls.

mRNA levels of marker genes in ROS, ER stress, glucose, and lipid metabolic pathways were examined by real-time PCR in hearts from 7-month-old mice (Table 1). Consistent with the increase in myocardial fructose content, mRNA levels of SDH were significantly increased in MHC-hAR (2.47-fold P<0.01), *Ppara^−/−^* (2.05-fold, P<0.01) and MHC-hAR/*Ppara^−/−^* (2.49-fold, P<0.001) compared with control mice. mRNA levels of Glut1and Glut4 were not changed in MHC-hAR mice, but were up-regulated by crossing MHC-hAR mice onto the *Ppara^−/−^* background. The increase in glucose uptake was associated with increased hexokinase 1 mRNA expression. PDK4 protein levels were decreased in all three genotypes (Figure 5A). The decrease of PDK4 protein usually correlates with greater glucose oxidation. FA oxidation related genes: CPT1 and AOX were significantly decreased in all three genotypes. We then checked expression of CD36 and lipoprotein lipase (LpL) in these mice to see whether decreased expression of FA transport and oxidation genes correlated with changes in FA uptake genes. Cardiac expression of LpL was not changed in these three groups of mice compared with WT mice. However, expression of CD36 was increased (2.32-fold, P<0.01) in MHC-hAR/*Ppara^−/−^* mice and unchanged in MHC-hAR and *Ppara^−/−^* mice. Taken together, these results suggest that crossing MHC-hAR on *Ppara^−/−^* background altered cardiac metabolic gene expression with increased glucose uptake and oxidation and decreased FA oxidation without a reduction in FA uptake.

**Table 1:** qRT-PCR analysis of heart tissue mRNA expression.

	Gene symbol	Control (n = 9)	MHC-hAR (n = 6)	PPARα^−/−^ (n = 10)	MHC-hAR/PPARα^−/−^ (n = 11)
Glucose Metabolism	SDH	1.00±0.29	2.47±0.55#	2.06±0.65#	2.48±0.37#
	Glut1	1.00±0.16	1.02±0.20^b^	2.22±0.46§ ^,^ b	1.75±0.30§
	Glut4	1.00±0.17	1.24±0.20^a^	1.73±0.31§	1.90±0.27§
	HK	1.00±0.56	2.33±1.01*	2.62±1.08#	4.25±1.14#
Lipid Metabolism	CD36	1.00±0.57	1.15±0.34^b^	1.22±0.24^b^	2.32±0.44#
	LPL	1.00±0.19	1.05±0.10	1.15±0.26	1.27±0.28
	CPT1	1.00±0.10	0.70±0.12# ^,^ a	0.47±0.07§	0.40±0.05§
	AOX	1.00±0.19	0.73±0.10# ^,^ a	0.48±0.08§	0.44±0.08§
Antioxidation and Apoptosis	SOD2	1.00±0.13	1.04±0.19	1.03±0.15	0.91±0.16
	BAX	1.00±0.07	1.07±0.37^a^	0.89±0.18^b^	1.66±0.58*
	CHOP	1.00±0.06	1.05±0.11^a^	1.15±0.15	1.37±0.20*

7-month old male mice after 16-hour fasting. Data were normalized to 18s rRNA. Values represent fold change relative to wild-type controls, which was set as 1. Data are shown as mean ± SD.

* P<0.05,

# P<0.01, and

§ P<0.001 compared with controls.

a P<0.05,

b P<0.01 compared with MHC-hAR/*Ppara^−/−^* mice.

### MHC-hAR expression increased cardiac glucose uptake on the Ppara^−/−^ background

We next determined whether reduction of *Ppara* expression altered cardiac glucose uptake in MHC-hAR/*Ppara^−/−^* mice. Plasma clearance for 2-deoxy-d-[3H]-glucose was not different between the genotypes. Cardiac glucose uptake was increased 2.1-fold in the MHC-hAR/*Ppara* ^−/−^ mice hearts compared with the controls (P<0.05; Figure S3A, B). This suggests that cardiac deletion of *Ppara* increased glucose uptake in MHC-hAR mice.

### MHC-hAR expression increased cardiac fructose levels on the Ppara^−/−^ background

Increased cardiac glucose uptake is likely to increase glucose entering the polyol pathway when AR is overexpressed. Furthermore, continued flux of sorbitol via SDH is likely to increase levels of fructose. To assess whether *Ppara* deficiency altered heart polyol pathway flux in MHC-hAR mice, we measured heart tissue fructose. Heart fructose levels were increased in MHC-hAR and MHC-hAR*/Ppara^−/−^* mice (P<0.05) compared to WT mice (Figure 5B). Fructose level was also increased in *Ppara^−/−^* hearts (P<0.05), indicating that deletion of *Ppara* increased glucose flux via the polyol pathway.

### MHC-hAR expression increased heart ceramide content on the Ppara^−/−^ background

**Figure 6:**
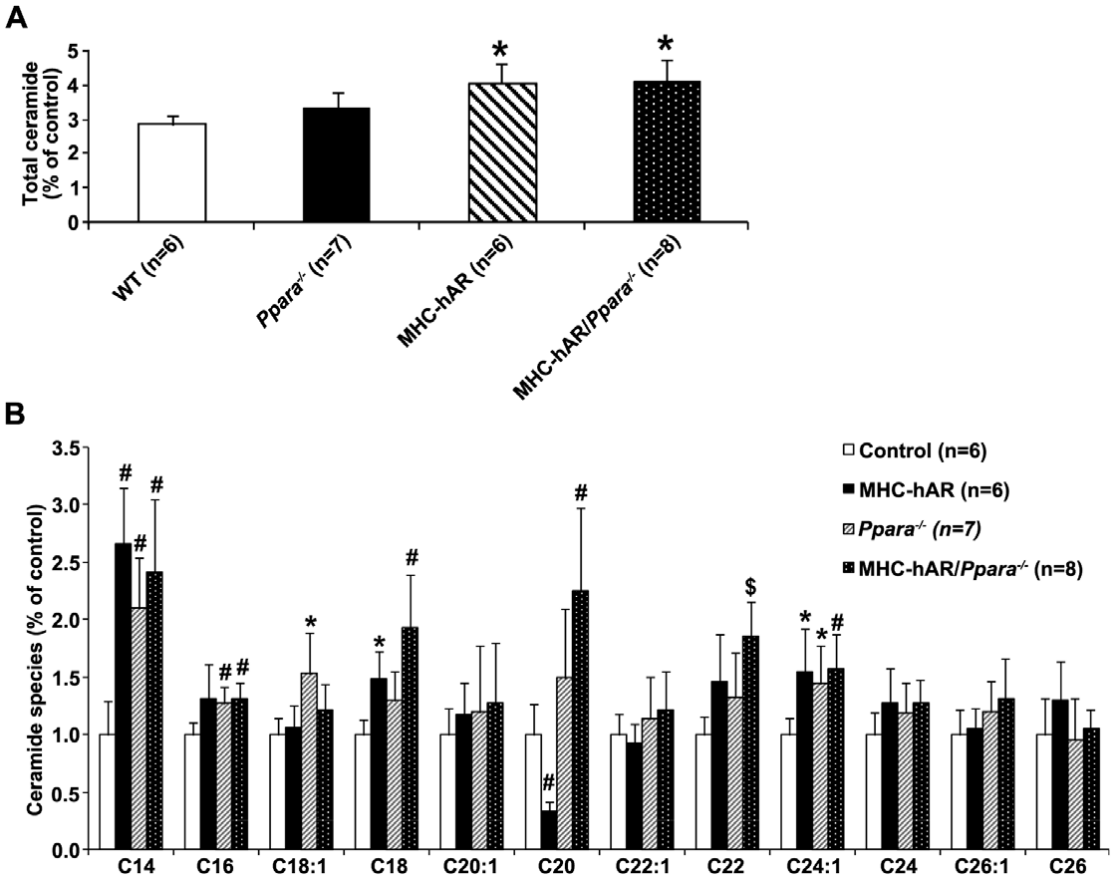
Cardiac ceramide in MHC-hAR/*Ppara* ^−/−^ mice. (**A**) Total ceramide and (**B**) individual ceramide species. Ceramide species data represent the content of each FA as a percentage of total ceramide and are shown as mean ± SD (n = 6–8 per group). *P<0.05, ^#^P<0.01 and ^§^P<0.001 versus littermate controls. ^a^ P<0.05 and ^b^P<0.01 versus MHC-hAR/*Ppara^−/−^* mice.

A surprising finding was that despite the reduction in mRNA levels of FA oxidation genes, mRNA levels of genes involved in heart lipid uptake were not decreased (Table 1). FA uptake/utilization mismatch could lead to accumulation of toxic lipids, which might have led to cardiac dysfunction. Levels of ceramide, a toxic lipid capable of inducing apoptosis, [26] were increased in MHC-hAR and MHC-hAR/*Ppara^−/−^* mice compared with control mice (Figure 6A). Toxic cardiac ceramide species, C24:1 and C24 ceramides, were increased in these hearts (Figure 6B).

### MHC-hAR expression increased cardiac apoptosis, ROS and fibrosis on the Ppara^−/−^ background

**Figure 7:**
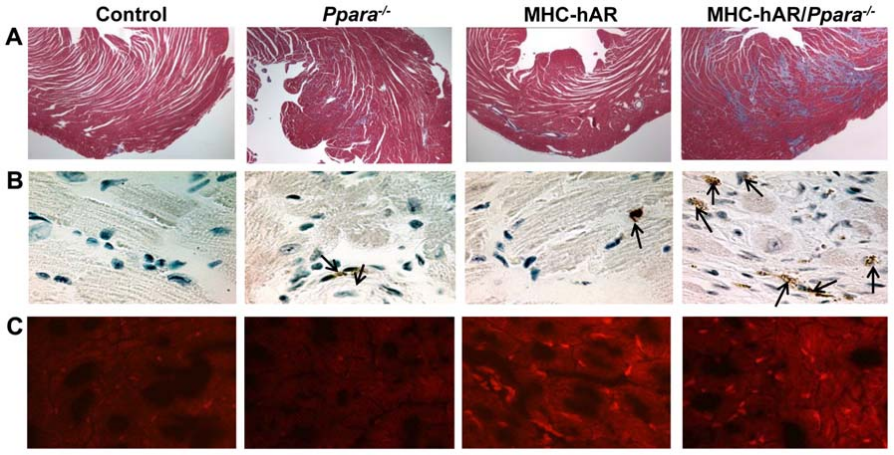
Increased cardiac fibrosis, apoptosis and ROS in MHC-hAR/*Ppara* ^−/−^ mice. (**A**) Cardiac fibrosis was detected using Masson's trichrome stain (original magnification, ×100) (**B**) Cardiac ventricular tissues were stained for DNA fragmentation by TUNEL protocol (original magnification, ×200). Apoptotic cardiomyocytes containing fragmented nuclear chromatin exhibited dark brown nuclear staining (arrows). (**C**) Histological analysis of heart tissues using dihydroethidium staining to detect ROS (original magnification, ×100).

Both gene expression and histology revealed the reasons for heart dysfunction with hAR expression. Most studies have linked increased flux via the polyol pathway to greater levels of ROS in tissue. [27], [28] Heart fibrosis and TUNEL-positive myocytes were slightly increased in MH-hAR and *Ppara^−/−^* mice but dramatically increased in MHC-hAR/*Ppara^−/−^* mice (Figure 7A and B). As anticipated, the heart tissue intracellular O_2_^−^ levels measured using dihydroethidium and fluorescence staining were increased in MHC-hAR mice and MHC-hAR/*Ppara^−/−^* (Figure 7C). The increase of TUNEL-positive myocytes was associated with increased expression of the apoptosis-related genes CHOP and Bax (Table 1 and Figure 5A)

## Discussion

There is a biochemical link between increased polyol pathway flux and diabetic complications, [12], [29], [30], [31] including microvascular damage to the retina, [10] kidney, [11] and nerves [32] and macrovascular damage. [14], [15] Our previous studies and those of others have shown that AR mediates cardiac ischemic injury both in diabetic and non-diabetic animals. [4], [5], [6], [13], [21], [33], [34] While the above studies have focused on the role of AR in using glucose as a substrate, others have shown that AR is an important component of antioxidant defenses involved in the removal and detoxification of reactive aldehydes generated by lipid peroxidation. [35], [36] Thus, the role of AR in tissue damage appears to depend on the model studied, the amount of AR expression, and the partial or complete level of AR deficiency. Our studies using a new transgenic mouse model showed the following: 1) AR expression to human-like levels caused heart dysfunction with aging. 2) Cardiomyocyte hAR expression increased ischemia/reperfusion injury. 3) When crossed onto a model of greater glucose oxidation, hAR expression led to more dysfunction. 4) The reasons for the heart dysfunction included greater accumulation of fructose and ceramide, more ROS production and increased apoptosis.

We created a mouse model with cardiac specific expression of human AR and used it as a tool to define the functional consequences of cardiac AR expression under physiological and pathological conditions. The first objective of this investigation was to determine whether cardiomyocyte expression of AR alters heart glucose and FA metabolism. Despite increased hAR and its downstream gene SDH, the genes involved in glucose and FA oxidation were not changed in 3 month-old MHC-hAR mice. Cardiac function in these mice was examined by echocardiography and did not differ from WT controls (Figure S4). These data show that in the normal physiological state the human AR transgene does not alter expression of genes involved in cardiomyocyte glucose and FA metabolism.

Data from clinical and mouse studies have shown an age-dependent decrease in myocardial FA utilization and relative increase in myocardial glucose uptake. [22], [23] Reduction of cardiac FA oxidation shifts the heart's energy source towards glucose use. [37] Recent studies have also shown that aging increases the expression and activities of AR and SDH in the heart and that the increases coupled with impaired substrate metabolism is linked to poor outcome after ischemic stress. [38] In our study, age-dependent changes in cardiac metabolism were detected in 15 month-old MHC-hAR mouse hearts. Expression of genes involved in both FA transport and oxidation, such as CPT1 and AOX, were decreased in these mice that had mild dilated cardiomyopathy. AR transgene expression did not alter expression of glucose transporter genes (Glut1 and Glut4), but reduced the expression of PDK4. Thus, aged MHC-hAR mice had cardiac dysfunction along with a shift towards decreased FA metabolism and increased glucose uptake.

Flux via AR increases under ischemic conditions, even in the absence of diabetes. [4], [5], [6], [13], [21], [33], [34] Studies using transgenic mice expressing human-relevant levels of AR (naturally higher than levels in mouse) revealed marked increases in ischemia/reperfusion injury in hearts from transgenic versus WT mice. [4] Consistent with the premise that injury was increased directly via AR, inhibitors of AR in these transgenic mice reduced ischemia/reperfusion injury, in part, by attenuating oxidative stress and apoptosis. [4], [21] Here we demonstrate that increased ischemia-reperfusion injury in cardiomyocyte specific hAR mice is linked to changes in apoptosis pathway genes. Here we demonstrate that increased ischemia-reperfusion injury in cardiomyocyte specific hAR mice is linked to changes in apoptosis pathway genes. Again despite data suggesting a pathological role for AR during ischemia–reperfusion, [4], [5], [6], [13], [21], [33], [34] some studies have shown contrary results. [35], [36] Although AR activity during ischemia was increased, cardioprotection with ARIs was not evident in their glucose perfused isolated rat heart ischemia-reperfusion model. [35], [36] Reasons for these contrasting results could, in part, be due to ischemia-reperfusion model-dependent variations and substrate availability.

Here we demonstrate that, in the setting of LAD ligation/reperfusion, MHC-hAR mice exhibited increased infarct size and poor functional recovery, and had decreased expression of *Ppara*. The cardiac role of PPARα is controversial because both *Ppara* knockout and *Ppara* overexpression lead to detrimental phenotypes in the mouse heart. [39], [40] Guellich et al. [41] showed that PPARα deficiency impairs cardiac function, in part, by causing oxidative damage to myosin. In another study, mice with cardiomyocyte-restricted knock out of the insulin receptor, when subjected to ischemia-reperfusion also exhibited repression of *Ppara* and key FA metabolism genes. These mice also had poor outcome after ischemia-reperfusion. [42] Our results are consistent with the above studies indicating that reduced PPARα in hAR mice during ischemia-reperfusion is detrimental to the heart.

We next sought to evaluate the metabolic and functional consequences of chronic suppression of FA oxidation in MHC-hAR heart. *Ppara^−/−^* mice have decreased FA oxidation and increased glucose oxidation in the heart. [24] In our study, cardiac metabolism genes were altered by performing a cross of MHC-hAR onto the *Ppara^−/−^* background. Glucose uptake was increased and PDK4 protein levels were decreased by this cross, consistent with greater glucose use. Surprisingly FA uptake genes (CD36 and LpL) did not decrease in concert with the genes for FA oxidation. The mechanism for the increase of CD36 in these mice is unclear. The mismatch between myocardial FA uptake and utilization genes is likely to result in FA accumulation and generation of cardiotoxic lipid species. Consistent with our line of reasoning, MHC-hAR/*Ppara^−/−^* hearts show higher levels of ceramide species. These ceramide species are potentially toxic molecules that have been linked to several forms of lipotoxic cardiomyopathy. [43], [44] It is possible that continued uptake of FA, especially palmitate, in the presence of reduced FA oxidation drove ceramide production in this model. Coincident with increased ceramide levels, MHC-hAR/*Ppara^−/−^* hearts show greater cardiomyocyte damage with more apoptosis, ROS and fibrosis. These results support our hypothesis that substantial decreases in FA utilization led to cardiac dysfunction in MHC-hAR/*Ppara^−/−^* hearts. Ceramide is an established cause of cellular apoptosis [45] and its reduction is associated with better insulin signaling, [46] reduced lipotoxicity, [41] and less cardiac dysfunction after cellular death. [47]

So far the most extensively documented roles of AR are in diabetic complications. Research on AR has focused on regulation of glucose metabolism. PPARα deficiency-mediated cardiac damage is associated with a shift in cardiac substrate utilization toward glucose, which is similar to clinical chronic ischemic heart disease. [48], [49], [50] The increases in cardiac SDH mRNA and fructose levels in *Ppara^−/−^* hearts suggest that reduced PPARα- mediated cardiac dysfunction is also associated with increased conversion of glucose to fructose by AR.

In summary, we show that cardiomyocyte expression of hAR plays a key role in the pathogenesis of cardiac dysfunction. Specifically, AR-mediates cardiac dysfunction in aging and in the setting of reduced FA and increased glucose uptake. Furthermore, we show that cardiomyocyte hAR is linked to increased injury in hearts after ischemia-reperfusion. Since increased flux via AR is pathogenic under these varying clinical conditions, inhibition of AR may be a new approach for the prevention and treatment of some forms of heart failure.

## Supporting Information

Figure S1 **Cardiac mRNA expression and echocardiographic measurement in 3-month old MHC-hAR mice.** (**A**) Cardiac mRNA expression. (**B–D**) Heart to body ratio and echocardiography results. Data are shown as mean ± SD (n = 6–8).
(PDF)

Figure S2 **Strategy for creating MHC-hAR/PPARα^−/−^ mice.**
(PDF)

Figure S3 **MHC-hAR expression increased cardiac glucose uptake in MHC-hAR/PPARα^−/−^ mice.** (**A**) Plasma clearance for 2-deoxy-d-[3H]-glucose and (**B**) cardiac glucose uptake in the MHC-hAR/PPARα−/− mice and wild type controls. Data are shown as mean ± SD. *P<0.05 compared with MHC -hAR mice.
(PDF)

Figure S4 **Echocardiography results from 3- to 13-month old mice.** Data are shown as mean ± SD (n = 6–13). **P<0.01 compared with MHC -hAR mice.
(PDF)

Table S1 **Plasma TG, TC, FFA and Glucose in 3- and 15-month old MHC-hAR mice.**
(PDF)

Table S2 **Plasma TG, TC, FFA and Glucose in 7-month old MHC-hAR and MHC-hAR/PPARα^−/−^ mice.**
(PDF)

## References

[1] Lionetti V, Stanley WC, Recchia FA (2011) Modulating fatty acid oxidation in heart failure. Cardiovasc Res 90: 202–209. 21289012 10.1093/cvr/cvr038 PMC3078800

[2] Okere IC, Young ME, McElfresh TA, Chess DJ, Sharov VG, et al (2006) Low carbohydrate/high-fat diet attenuates cardiac hypertrophy, remodeling, and altered gene expression in hypertension. Hypertension 48: 1116–1123. 17060511 10.1161/01.HYP.0000248430.26229.0f

[3] Kloner RA, Nesto RW (2008) Glucose-insulin-potassium for acute myocardial infarction: continuing controversy over cardioprotection. Circulation 117: 2523–2533. 18474824 10.1161/CIRCULATIONAHA.107.697979

[4] Hwang YC, Kaneko M, Bakr S, Liao H, Lu Y, et al (2004) Central role for aldose reductase pathway in myocardial ischemic injury. FASEB J 18: 1192–1199. 15284219 10.1096/fj.03-1400com

[5] Hwang YC, Sato S, Tsai JY, Yan S, Bakr S, et al (2002) Aldose reductase activation is a key component of myocardial response to ischemia. FASEB J 16: 243–245. 11772943 10.1096/fj.01-0368fje

[6] Tracey WR, Magee WP, Ellery CA, MacAndrew JT, Smith AH, et al (2000) Aldose reductase inhibition alone or combined with an adenosine A(3) agonist reduces ischemic myocardial injury. Am J Physiol Heart Circ Physiol 279: H1447–1452. 11009428 10.1152/ajpheart.2000.279.4.H1447

[7] Trueblood N, Ramasamy R (1998) Aldose reductase inhibition improves altered glucose metabolism of isolated diabetic rat hearts. Am J Physiol 275: H75–83. 9688898 10.1152/ajpheart.1998.275.1.H75

[8] Hers HG (1956) [The mechanism of the transformation of glucose in fructose in the seminal vesicles]. Biochim Biophys Acta 22: 202–203. 13373872 10.1016/0006-3002(56)90247-5

[9] Gabbay KH (1973) The sorbitol pathway and the complications of diabetes. N Engl J Med 288: 831–836. 4266466 10.1056/NEJM197304192881609

[10] Kinoshita JH, Fukushi S, Kador P, Merola LO (1979) Aldose reductase in diabetic complications of the eye. Metabolism 28: 462–469. 45423 10.1016/0026-0495(79)90057-x

[11] Dunlop M (2000) Aldose reductase and the role of the polyol pathway in diabetic nephropathy. Kidney Int Suppl 77: S3–12. 10997684 10.1046/j.1523-1755.2000.07702.x

[12] Williamson JR, Chang K, Frangos M, Hasan KS, Ido Y, et al (1993) Hyperglycemic pseudohypoxia and diabetic complications. Diabetes 42: 801–813. 8495803 10.2337/diab.42.6.801

[13] Ramasamy R, Oates PJ, Schaefer S (1997) Aldose reductase inhibition protects diabetic and nondiabetic rat hearts from ischemic injury. Diabetes 46: 292–300. 9000707 10.2337/diab.46.2.292

[14] Vikramadithyan RK, Hu Y, Noh HL, Liang CP, Hallam K, et al (2005) Human aldose reductase expression accelerates diabetic atherosclerosis in transgenic mice. J Clin Invest 115: 2434–2443. 16127462 10.1172/JCI24819 PMC1190371

[15] Vedantham S, Noh H, Ananthakrishnan R, Son N, Hallam K, et al (2011) Human aldose reductase expression accelerates atherosclerosis in diabetic apolipoprotein E−/− mice. Arterioscler Thromb Vasc Biol 31: 1805–1813. 21636809 10.1161/ATVBAHA.111.226902 PMC3278231

[16] Markus HB, Raducha M, Harris H (1983) Tissue distribution of mammalian aldose reductase and related enzymes. Biochem Med 29: 31–45. 6404249 10.1016/0006-2944(83)90051-0

[17] Srivastava S, Vladykovskaya E, Barski OA, Spite M, Kaiserova K, et al (2009) Aldose reductase protects against early atherosclerotic lesion formation in apolipoprotein E-null mice. Circ Res 105: 793–802. 19729598 10.1161/CIRCRESAHA.109.200568 PMC3548455

[18] Son NH, Yu S, Tuinei J, Arai K, Hamai H, et al (2010) PPARgamma-induced cardiolipotoxicity in mice is ameliorated by PPARalpha deficiency despite increases in fatty acid oxidation. J Clin Invest 120: 3443–3454. 20852389 10.1172/JCI40905 PMC2947216

[19] Aleshin A, Ananthakrishnan R, Li Q, Rosario R, Lu Y, et al (2008) RAGE modulates myocardial injury consequent to LAD infarction via impact on JNK and STAT signaling in a murine model. Am J Physiol Heart Circ Physiol 294: H1823–1832. 18245563 10.1152/ajpheart.01210.2007

[20] Vivaldi MT, Kloner RA, Schoen FJ (1985) Triphenyltetrazolium staining of irreversible ischemic injury following coronary artery occlusion in rats. Am J Pathol 121: 522–530. 2416222 PMC1887916

[21] Ananthakrishnan R, Kaneko M, Hwang YC, Quadri N, Gomez T, et al (2009) Aldose reductase mediates myocardial ischemia-reperfusion injury in part by opening mitochondrial permeability transition pore. Am J Physiol Heart Circ Physiol 296: H333–341. 19060123 10.1152/ajpheart.01012.2008 PMC2643894

[22] Kates AM, Herrero P, Dence C, Soto P, Srinivasan M, et al (2003) Impact of aging on substrate metabolism by the human heart. J Am Coll Cardiol 41: 293–299. 12535825 10.1016/s0735-1097(02)02714-6

[23] Hyyti OM, Ledee D, Ning XH, Ge M, Portman MA (2010) Aging impairs myocardial fatty acid and ketone oxidation and modifies cardiac functional and metabolic responses to insulin in mice. Am J Physiol Heart Circ Physiol 299: H868–875. 20601465 10.1152/ajpheart.00931.2009 PMC2944494

[24] Campbell FM, Kozak R, Wagner A, Altarejos JY, Dyck JR, et al (2002) A role for peroxisome proliferator-activated receptor alpha (PPARalpha) in the control of cardiac malonyl-CoA levels: reduced fatty acid oxidation rates and increased glucose oxidation rates in the hearts of mice lacking PPARalpha are associated with higher concentrations of malonyl-CoA and reduced expression of malonyl-CoA decarboxylase. J Biol Chem 277: 4098–4103. 11734553 10.1074/jbc.M106054200

[25] Karbowska J, Kochan Z, Smolenski RT (2003) Peroxisome proliferator-activated receptor alpha is downregulated in the failing human heart. Cell Mol Biol Lett 8: 49–53. 12655356

[26] Crowder CM (2009) Cell biology. Ceramides–friend or foe in hypoxia? Science 324: 343–344. 19372418 10.1126/science.1173278 PMC3753222

[27] Obrosova IG, Pacher P, Szabo C, Zsengeller Z, Hirooka H, et al (2005) Aldose reductase inhibition counteracts oxidative-nitrosative stress and poly(ADP-ribose) polymerase activation in tissue sites for diabetes complications. Diabetes 54: 234–242. 15616034 10.2337/diabetes.54.1.234 PMC2756473

[28] Chung SS, Ho EC, Lam KS, Chung SK (2003) Contribution of polyol pathway to diabetes-induced oxidative stress. J Am Soc Nephrol 14: S233–236. 12874437 10.1097/01.asn.0000077408.15865.06

[29] Obrosova IG, Minchenko AG, Vasupuram R, White L, Abatan OI, et al (2003) Aldose reductase inhibitor fidarestat prevents retinal oxidative stress and vascular endothelial growth factor overexpression in streptozotocin-diabetic rats. Diabetes 52: 864–871. 12606532 10.2337/diabetes.52.3.864

[30] Collier A, Small M (1991) The role of the polyol pathway in diabetes mellitus. Br J Hosp Med 45: 38–40. 1901236

[31] Ramasamy R, Oates PJ (2003) Aldose reduction and vascular stress. In: Marso SP, Stern DM, editors. Textbook of Diabetes Mellitus and Cardiovascular Disease. Philadelphia: Lippincott Williams & Wilkins. pp. 55–74.

[32] Gabbay KH, Merola LO, Field RA (1966) Sorbitol pathway: presence in nerve and cord with substrate accumulation in diabetes. Science 151: 209–210. 5907911 10.1126/science.151.3707.209

[33] Iwata K, Matsuno K, Nishinaka T, Persson C, Yabe-Nishimura C (2006) Aldose reductase inhibitors improve myocardial reperfusion injury in mice by a dual mechanism. J Pharmacol Sci 102: 37–46. 16936455 10.1254/jphs.fp0060218

[34] Li Q, Hwang YC, Ananthakrishnan R, Oates PJ, Guberski D, et al (2008) Polyol pathway and modulation of ischemia-reperfusion injury in Type 2 diabetic BBZ rat hearts. Cardiovasc Diabetol 7: 33. 18957123 10.1186/1475-2840-7-33 PMC2584021

[35] Kaiserova K, Srivastava S, Hoetker JD, Awe SO, Tang XL, et al (2006) Redox activation of aldose reductase in the ischemic heart. J Biol Chem 281: 15110–15120. 16567803 10.1074/jbc.M600837200

[36] Shinmura K, Bolli R, Liu SQ, Tang XL, Kodani E, et al (2002) Aldose reductase is an obligatory mediator of the late phase of ischemic preconditioning. Circ Res 91: 240–246. 12169650 10.1161/01.res.0000029970.97247.57

[37] Kolwicz SC Jr, Tian R (2011) Glucose metabolism and cardiac hypertrophy. Cardiovasc Res 90: 194–201. 21502371 10.1093/cvr/cvr071 PMC3078804

[38] Ananthakrishnan R, Li Q, Gomes T, Schmidt AM, Ramasamy R (2011) Aldose reductase pathway contributes to vulnerability of aging myocardium to ischemic injury. Exp Gerontol 46: 762–767. 21600277 10.1016/j.exger.2011.05.001 PMC3144997

[39] Finck BN, Lehman JJ, Leone TC, Welch MJ, Bennett MJ, et al (2002) The cardiac phenotype induced by PPARalpha overexpression mimics that caused by diabetes mellitus. J Clin Invest 109: 121–130. 11781357 10.1172/JCI14080 PMC150824

[40] Smeets PJ, Teunissen BE, Willemsen PH, van Nieuwenhoven FA, Brouns AE, et al (2008) Cardiac hypertrophy is enhanced in PPAR alpha−/− mice in response to chronic pressure overload. Cardiovasc Res 78: 79–89. 18187461 10.1093/cvr/cvn001

[41] Guellich A, Damy T, Lecarpentier Y, Conti M, Claes V, et al (2007) Role of oxidative stress in cardiac dysfunction of PPARalpha−/− mice. Am J Physiol Heart Circ Physiol 293: H93–H102. 17369471 10.1152/ajpheart.00037.2007

[42] Sena S, Hu P, Zhang D, Wang X, Wayment B, et al (2009) Impaired insulin signaling accelerates cardiac mitochondrial dysfunction after myocardial infarction. J Mol Cell Cardiol 46: 910–918. 19249310 10.1016/j.yjmcc.2009.02.014 PMC2683200

[43] Park TS, Hu Y, Noh HL, Drosatos K, Okajima K, et al (2008) Ceramide is a cardiotoxin in lipotoxic cardiomyopathy. J Lipid Res 49: 2101–2112. 18515784 10.1194/jlr.M800147-JLR200 PMC2533410

[44] Chiu HC, Kovacs A, Ford DA, Hsu FF, Garcia R, et al (2001) A novel mouse model of lipotoxic cardiomyopathy. J Clin Invest 107: 813–822. 11285300 10.1172/JCI10947 PMC199569

[45] Radin NS (2003) Killing tumours by ceramide-induced apoptosis: a critique of available drugs. Biochem J 371: 243–256. 12558497 10.1042/BJ20021878 PMC1223313

[46] Major CD, Gao ZY, Wolf BA (1999) Activation of the sphingomyelinase/ceramide signal transduction pathway in insulin-secreting beta-cells: role in cytokine-induced beta-cell death. Diabetes 48: 1372–1380. 10389841 10.2337/diabetes.48.7.1372

[47] Holland WL, Miller RA, Wang ZV, Sun K, Barth BM, et al (2011) Receptor-mediated activation of ceramidase activity initiates the pleiotropic actions of adiponectin. Nat Med 17: 55–63. 21186369 10.1038/nm.2277 PMC3134999

[48] Allard MF, Schonekess BO, Henning SL, English DR, Lopaschuk GD (1994) Contribution of oxidative metabolism and glycolysis to ATP production in hypertrophied hearts. Am J Physiol 267: H742–750. 8067430 10.1152/ajpheart.1994.267.2.H742

[49] Bishop SP, Altschuld RA (1970) Increased glycolytic metabolism in cardiac hypertrophy and congestive failure. Am J Physiol 218: 153–159. 4243400 10.1152/ajplegacy.1970.218.1.153

[50] Kagaya Y, Kanno Y, Takeyama D, Ishide N, Maruyama Y, et al (1990) Effects of long-term pressure overload on regional myocardial glucose and free fatty acid uptake in rats. A quantitative autoradiographic study. Circulation 81: 1353–1361. 2180593 10.1161/01.cir.81.4.1353

