# Supplementary figures and images for: Cardiomyocyte Aldose Reductase Causes Heart Failure and Impairs Recovery from Ischemia
Source: PLoS One. 2012 Sep 27;7(9):e46549. doi: 10.1371/journal.pone.0046549 (PMC3459912; doi:10.1371/journal.pone.0046549)

## Supplementary Figure S1

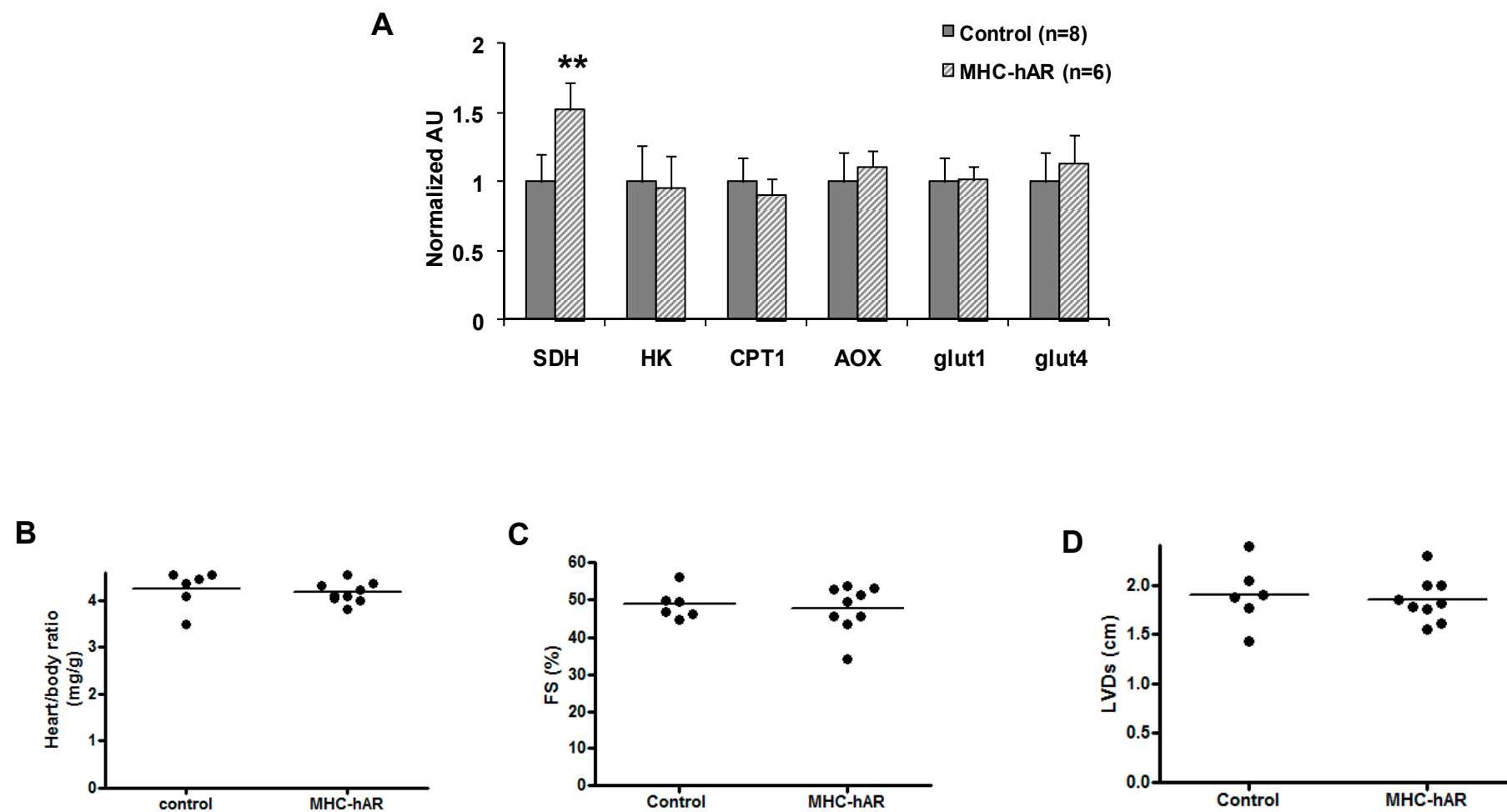

Supplement: Figure S1 — Cardiac mRNA expression and echocardiographic measurement in 3-month old MHC-hAR mice. (A) Cardiac mRNA expression. (B–D) Heart to body ratio and echocardiography results. Data are shown as mean ± SD (n = 6–8). (PDF) [file pone.0046549.s001.pdf]

Supplementary Figure S2

## Strategy for mouse breeding

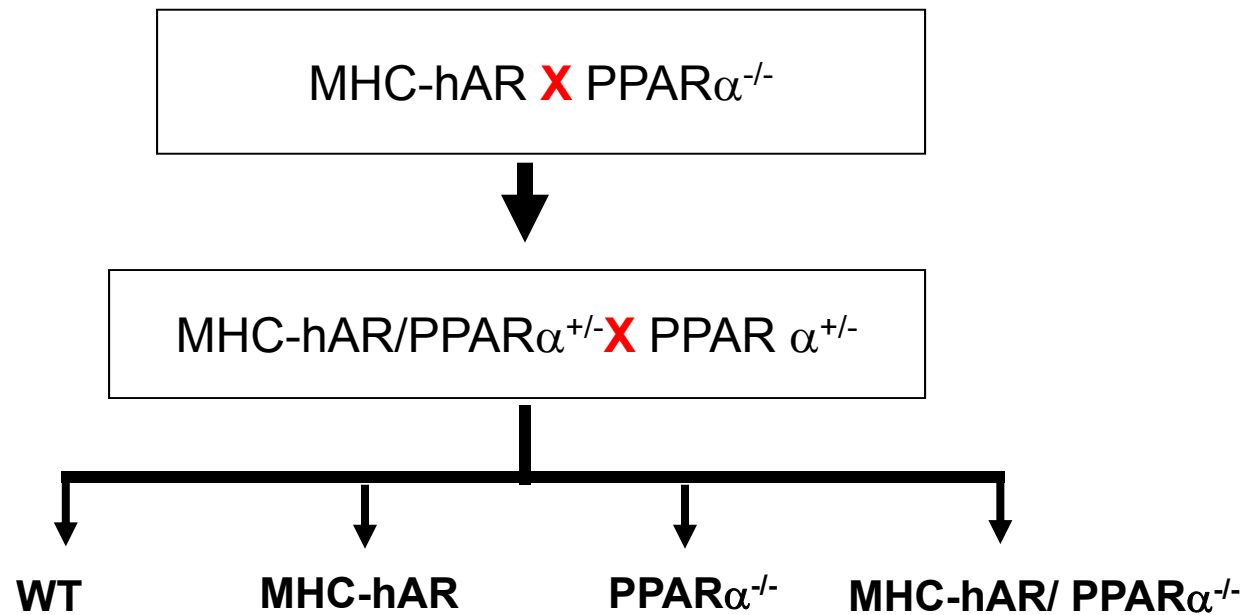

Supplement: Figure S2 — Strategy for creating MHC-hAR/PPARα−/− mice. (PDF) [file pone.0046549.s002.pdf]

Supplementary Figure S3

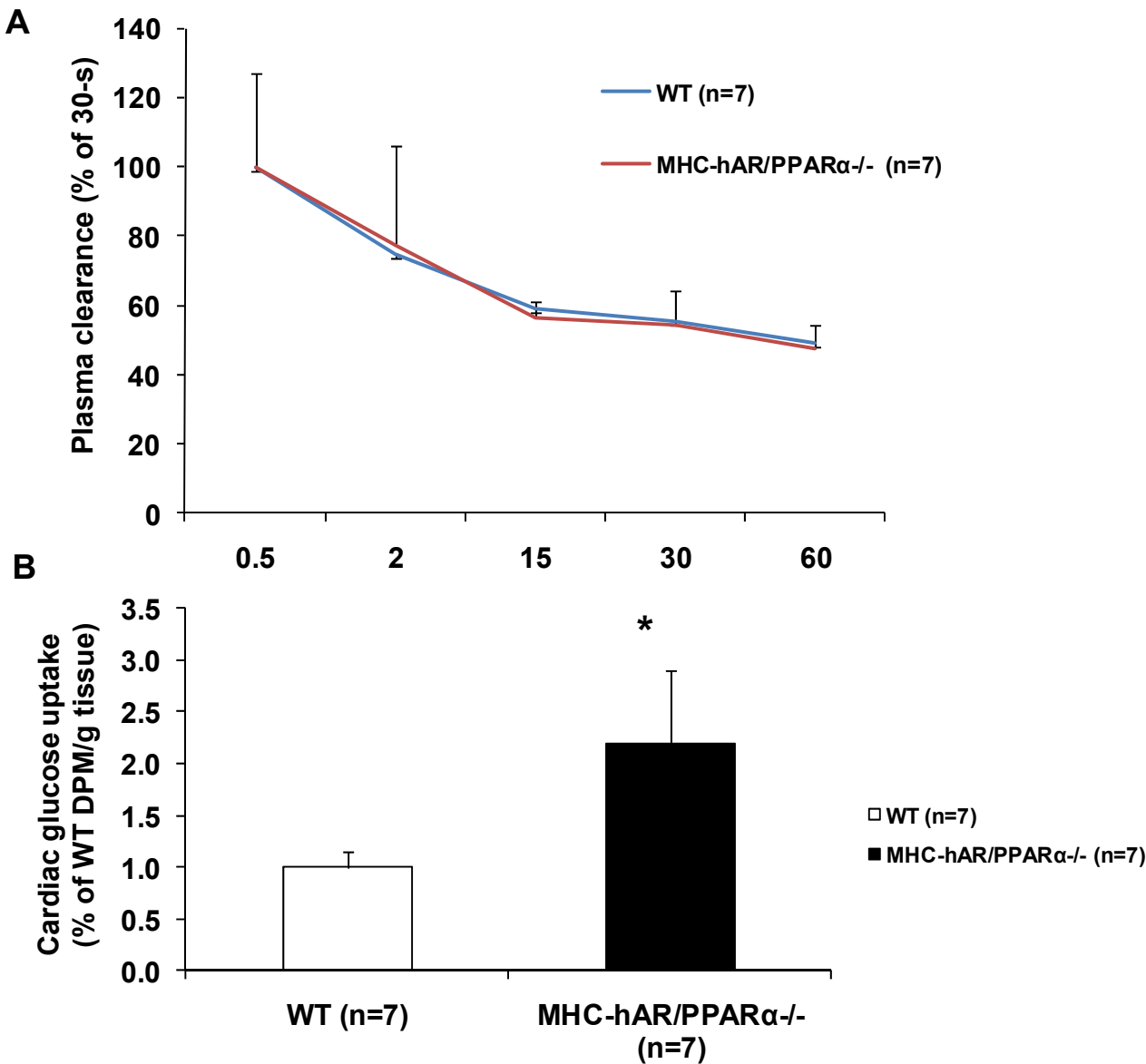

Supplement: Figure S3 — MHC-hAR expression increased cardiac glucose uptake in MHC-hAR/PPARα−/− mice. (A) Plasma clearance for 2-deoxy-d-[3H]-glucose and (B) cardiac glucose uptake in the MHC-hAR/PPARα−/− mice and wild type controls. Data are shown as mean ± SD. *P<0.05 compared with MHC -hAR mice. (PDF) [file pone.0046549.s003.pdf]

Supplementary Figure S4

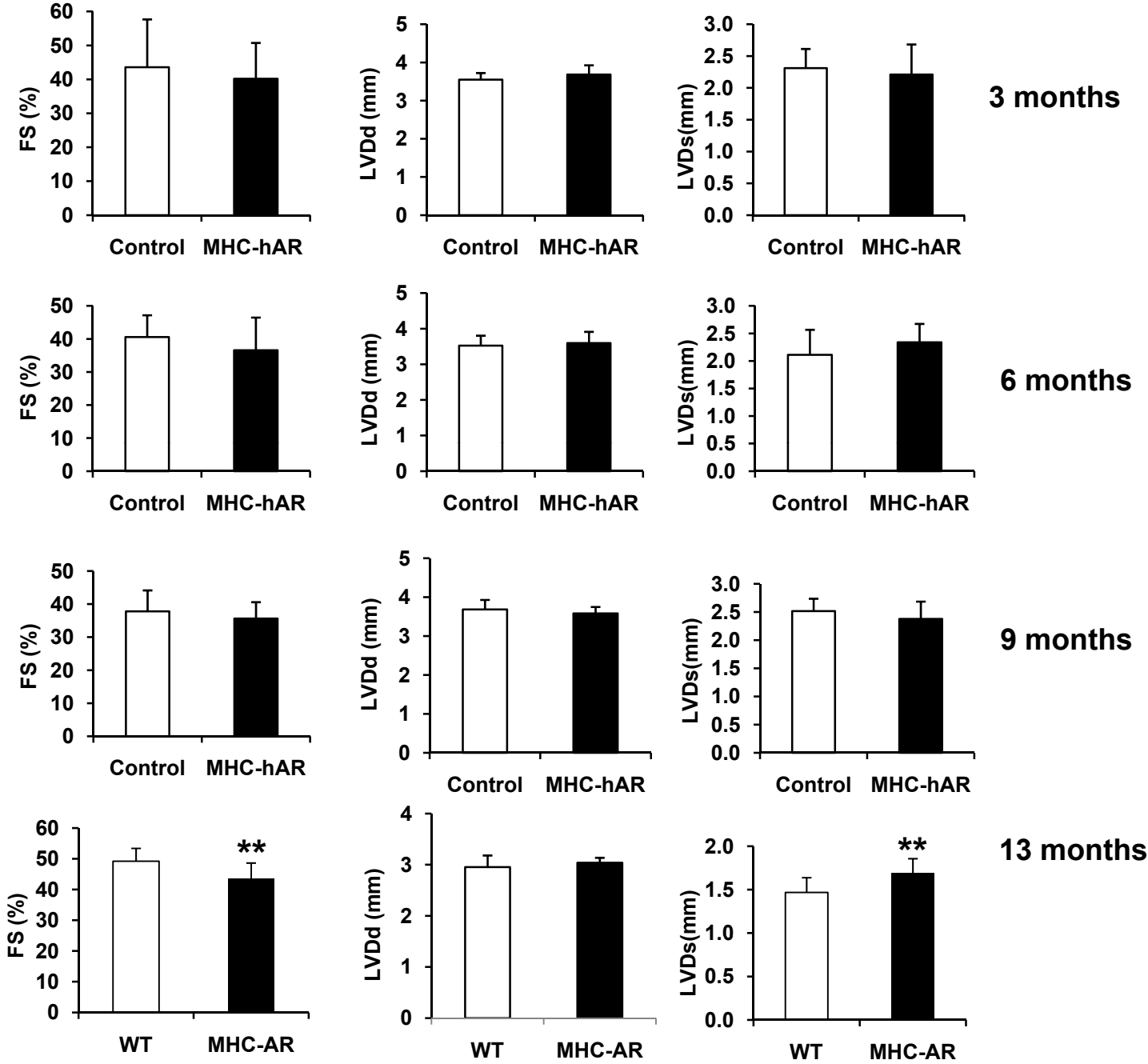

Supplement: Figure S4 — Echocardiography results from 3- to 13-month old mice. Data are shown as mean ± SD (n = 6–13). **P<0.01 compared with MHC -hAR mice. (PDF) [file pone.0046549.s004.pdf]
