# Supplementary material for: Cardiomyocyte Aldose Reductase Causes Heart Failure and Impairs Recovery from Ischemia
Source: PLoS One. 2012 Sep 27;7(9):e46549. doi: 10.1371/journal.pone.0046549 (PMC3459912; doi:10.1371/journal.pone.0046549)
Supplement: Table S1 — Plasma TG, TC, FFA and Glucose in 3- and 15-month old MHC-hAR mice. (PDF) [file pone.0046549.s005.pdf]

Supplementary Table S1

	3-month		13-month	
	Control (n=8)	MHC-hAR (n=11)	Control (n=14)	MHC-hAR (n=14)
FFA (mole/L)	0.96 ± 0.12	0.94 ± 0.10	0.40 ± 0.06	0.42± 0.09
TG (mg/dl)	68.6 ± 8.70	74.4± 10.8	140.0 ± 50.4	132.2 ± 31.2
TC (mg/dl)	84.5 ± 6.4	82.9 ± 9.0	126.6± 18.7	129.6 ± 11.6
Glucose (mg/dl)	135.6 ± 9.6	137.0 ± 9.1	155.7 ± 31.1	149.7 ± 18.9

Male mice with 6h fasting. Data are shown as mean (± S.D.).
