# Supplementary material for: Cardiomyocyte Aldose Reductase Causes Heart Failure and Impairs Recovery from Ischemia
Source: PLoS One. 2012 Sep 27;7(9):e46549. doi: 10.1371/journal.pone.0046549 (PMC3459912; doi:10.1371/journal.pone.0046549)
Supplement: Table S2 — Plasma TG, TC, FFA and Glucose in 7-month old MHC-hAR and MHC-hAR/PPARα−/− mice. (PDF) [file pone.0046549.s006.pdf]

Supplementary Table S2

	Control (n=6)	MHC-hAR (n=7)	PPAR$\alpha^{-/-}$ (n=8)	MHC-hAR/PPAR$\alpha^{-/-}$ (n=8)
FFA (mole/L)	1.07 ± 0.09	1.12 ± 0.11	1.40 ± 0.25*	1.29 ± 0.48
TG (mg/dl)	64.9 ± 8.70	63.8 ± 17.8	60.4 ± 9.6	60.7 ± 15.4
TC (mg/dl)	87.7 ± 16.2	99.7 ± 5.8	105.5 ± 10.6	107.2 ± 18.5
Glucose (mg/dl)	140.8 ± 32.7	116.8 ± 17.9	123.0 ± 15.5	113.3 ± 20.6

Male mice with 6h fasting. Data are shown as mean (± S.D.). *$P < 0.05$ *vs*. control mice.
